# Intelligent Smart Marine Autonomous Surface Ship Decision System Based on Improved PPO Algorithm

**DOI:** 10.3390/s22155732

**Published:** 2022-07-31

**Authors:** Wei Guan, Zhewen Cui, Xianku Zhang

**Affiliations:** Navigation College, Dalian Maritime University, Dalian 116026, China; cuizhewen123@dlmu.edu.cn (Z.C.); zhangxk@dlmu.edu.cn (X.Z.)

**Keywords:** decision-making, deep reinforcement learning, Nomoto, PPO, SMASS

## Abstract

With the development of artificial intelligence technology, the behavior decision-making of an intelligent smart marine autonomous surface ship (SMASS) has become particularly important. This research proposed local path planning and a behavior decision-making approach based on improved Proximal Policy Optimization (PPO), which could drive an unmanned SMASS to the target without requiring any human experiences. In addition, a generalized advantage estimation was added to the loss function of the PPO algorithm, which allowed baselines in PPO algorithms to be self-adjusted. At first, the SMASS was modeled with the Nomoto model in a simulation waterway. Then, distances, obstacles, and prohibited areas were regularized as rewards or punishments, which were used to judge the performance and manipulation decisions of the vessel Subsequently, improved PPO was introduced to learn the action–reward model, and the neural network model after training was used to manipulate the SMASS’s movement. To achieve higher reward values, the SMASS could find an appropriate path or navigation strategy by itself. After a sufficient number of rounds of training, a convincing path and manipulation strategies would likely be produced. Compared with the proposed approach of the existing methods, this approach is more effective in self-learning and continuous optimization and thus closer to human manipulation.

## 1. Introduction and Background

Since the 1970s, the combination of robot technologies and vehicles has led to the emergence of drones, unmanned vehicles, and unmanned ships [1]. Among them, a ship sailing on the sea is seriously affected by wind and surges. The decision-making and path planning of intelligent ships have been considered significant academic problems. Ships are generally under-actuated due to their large tonnage, slow speed, and relatively weak power. The autonomous navigation of ships has to meet huge inertia and complex navigation rules; therefore, the requirements for smart ships are much higher than those for unmanned vehicles. A ship operator faces many challenges, including those associated with the dynamic environment, insufficient power, and uncertainties in perception. According to the report of the International Maritime Organization, more than 80 percent of maritime accidents are caused by misunderstandings of the situation and by human error in decision-making resulting from failure to comply with the International Regulations for Preventing Collisions at Sea (COLREGs). Therefore, artificial intelligence for ship navigation is considered very difficult, and its core functions are path planning and intelligent decision-making.

Ship intelligent decision-making can be divided into two types: path planning and obstacle avoidance. One is the traditional model-based obstacle avoidance algorithm. For many years, the A* algorithm was the dominant approach in relevant research. A Swiss boat named Avalon was capable of generating a persuasive path to a given destination and avoiding both static and dynamic obstacles based on the A* algorithm [2]. Several heuristic function values of the current path grid are compared by the A* algorithm to gradually determine the next path grid, which can accurately avoid obstacles. However, when there were multiple minimum values, the optimal path could not be searched by the A* algorithm. Sudden obstacles would make the ship fall into the local optimum. Zhang et al. improved the Rapidly Exploring Random Tree (RRT) algorithm so that the convergence rate of the algorithm was significantly improved [3]. However, the path was randomly selected by the RRT algorithm, and the probability of encountering narrow channels was low. It was not appropriate to navigate in a narrow channel or to face multiple static obstacles. An ant colony optimization (ACO) and a clustering-based algorithm were proposed to settle the path planning of the USV by Liu et al. [4]. The improved ant colony optimization was used to adaptively select the appropriate search range, and the smoothing mechanism was used to adjust the path to achieve global path planning. An improved artificial potential field method (APF) was proposed by Shaorong Xie et al. The problem of USV falling into the unreachable local optimal target could be improved by this method, but there were still problems such as the poor accuracy of the algorithm and falling into local optimum in complex environments [5]. The gravitational field and repulsion field functions were required to be set separately; thus, this method does not apply to any environment. A new artificial potential field (APF) method was improved by Hongguang Lv et al. Different from the method proposed by Shaorong Xie et al., the new modified repulsive potential field function and the corresponding virtual force were introduced in the algorithm [6]. Appropriate functionality and security requirements were added to the corresponding virtual force to ensure compliance with the International Regulations for Preventing Collisions at Sea (COLREGs). However, with the complexity of modern maritime systems, a complete collision avoidance model is difficult to establish in many path planning and navigation problems. In most model-based algorithms, uncertainty is difficult to predict in practical applications.

Another is a model-free reinforcement learning algorithm that learns optimal strategies by interacting with the environment. At present, the development of artificial intelligence technology, especially reinforcement learning, provides a new possibility to satisfy the requirements of the path planning of intelligent ships. Reinforcement learning has attracted extensive attention in recent years, which emphasizes the learning of agents from the environment to behavior mapping and seeks the most correct or best action decision by maximizing value functions. A ship path planning algorithm based on Q learning was proposed by Chen C. et al. Combined with the ship mathematical model, the USV could obtain a higher reward value by learning the action-value function [7]. However, the reinforcement learning algorithm had an insufficient perception of the external environment, and the action state information was difficult to be searched. In addition, the experimental environment was too simple, without considering the decision problem of complex multi-obstacles. An algorithm was proposed by Everett et al. that generates an appropriate collision-free path even when the number of dynamic obstacles is changed by using Long short-term memory (LSTM) [8]. A Deep-Q-Learning (DQN) algorithm linking perception and decision-making was proposed by Jingwei Zhang et al. The algorithm could acquire external images by depth camera information and extracts image features as inputs of DQN [9]. Decision problems can be solved by this algorithm, but the use of a depth camera and convolution network makes the calculation huge. Moreover, when sailing in harsh sea conditions, the depth camera could not be effectively put into use, and the method would be not convincing. A DQN-based path planning obstacle avoidance algorithm was proposed by Haiqing Shen et al. The algorithm could be successfully simulated with human experience and International Regulations for Preventing Collisions at Sea [10]. However, the DQN algorithm has an overestimation problem, and an unmanned surface vessel (USV) is prone to action selection error in a more complex environment. An algorithm combining Deep-Q-Learning (DQN) and the artificial potential field (APF) was proposed by Lingyu Li et al., which was used for USV path planning [11]. This algorithm made deep reinforcement learning more purposeful in the early stage of training and had a faster convergence effect. However, the method based on Q-learning seemed inadequate in solving the problem of continuous action. A Multi-Experience Library Framework was proposed by Zijian Hu et al. for Unmanned Aerial Vehicle (UAV) autonomous motion planning. The algorithm generated expert experience by model predictive control and simulated annealing [12]. When applying this algorithm to a complex unknown simulation environment constructed based on the parameters of the real UAV, the training experiment results showed that the novel Deep reinforcement learning (DRL) algorithm led to a performance improvement exceeding twenty percent, as compared to the state-of-the-art Deep Deterministic Policy Gradient (DDPG). DDPG has a slightly better decision-making effect than value-based learning algorithms in complex environments. Choosing the maximum probability action in each step under continuous action can make calculation much simpler. A new quantitative risk assessment method was proposed by Do-Hyun Chun et al. In the calculation of collision risk (CR), the distance closest point of approach (DCPA) and time closest point of approach (TCPA) were determined by ship length and ship speed [13]. This algorithm could take the collision risk assessment CR as one of the inputs of the neural network, but the experiment was too simple to generalize. An obstacle avoidance method based on the combination of PPO and the Line of Sight (LOS) guidance system was proposed by Luman Zhao et al. This algorithm could ensure that the ship moves along the predetermined path and avoids collision with the moving ship. Due to the limitation of the LOS algorithm, this experiment cannot avoid collision in a complex environment [14]. An improved DQN algorithm was proposed by Xinli Xu et al. The network weight was set by them to slowly approach the current value; in other words, the target network would approach the evaluation network gradually [15]. It could reduce the correlation between the current value function and the target value function to some extent. In addition, the reward function of the algorithm made the USV alter different angles, and the reward value was also different. However, the algorithm based on value learning was still overestimated, and the problems of static obstacles and generalization were not considered in the experiment. A distributed sensor-level collision avoidance policy for multi-robot systems was proposed by Pinxin Long et al., which could directly map raw sensor measurements to an agent’s steering commands in terms of movement velocity [16]. This experiment verified the learned sensor-level collision avoidance strategy in various simulation scenarios and conducted a comprehensive performance evaluation. This experiment also demonstrated that the learned policy could be well generalized to new scenarios that did not appear in the entire training period, including navigating a heterogeneous group of robots and a large-scale scenario with 100 robots. Pinxin Long et al.’s experiment had a strong generalization ability, which is worth learning.

Based on the above research, an intelligent smart marine autonomous surface ship (SMASS) decision system based on an improved PPO algorithm was proposed in this paper. The main contributions of this article were as follows:

- An intelligent SMASS decision-making system based on the Proximal Policy Optimization (PPO) algorithm was proposed in this paper, which could make the critic network and action network converge faster.
- Through the Gazebo simulation environment, sensors such as laser radar and navigation radar were used to obtain external environmental information. Intelligent SMASS could make complex path planning decisions in different environments. After the training, if unknown obstacles are placed on the map, the intelligent ship could still successfully avoid obstacles.
- The Nomoto model was brought into the training of this experiment. Training the model could meet the needs of practical engineering.

The rest of this paper is organized as follows. Section 2 introduces the composition of the intelligent SMASS system, ship mathematical model, and COLREGs. Section 3 introduces a deep reinforcement learning algorithm and improved Proximal Policy Optimization (PPO) algorithm. Section 4 mainly introduces the reward function setting and network setting. Section 5 mainly introduces the design of the Gazebo simulation and the analysis of experimental results. Section 6 is the summary of this paper and the future research planning.

## 2. Intelligent Ship Decision System and Ship Mathematical Model

**Figure 1:**
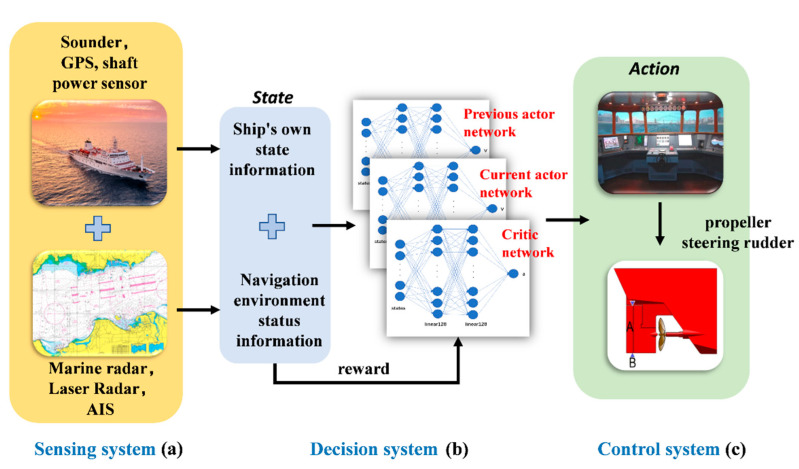
Intelligent ship navigation system: (**a**) the sensing part, (**b**) the decision part, and (**c**) the control part.

In building a complete set of the intelligent smart marine autonomous surface ship (SMASS) decision-making systems, it was necessary to clarify the components of the system, the functions of each part, and the relationship between different parts [17]. There are three parts included in the intelligent smart marine autonomous surface ship (SMASS) decision-making system, namely, the sensing part, the decision-making part, and the control part, as shown in Figure 1.

### 2.1. Intelligent Ship Decision System

The sensing part is divided into the SMASS’s own state information and navigation environment information. The sensing part is mainly composed of navigation radar, laser radar, GPS, shaft power sensor, bathymeter, speed sensor, and AIS. The SMASS’s own state information includes the SMASS’s course, speed, position, oil consumption remaining, propeller speed, and hull structure strength [18]. Navigation environment information includes other ship heading speed TCPA, DCPA, hydrological information, velocity, channel depth, meteorological information (temperature, humidity, wind direction, wind speed), electronic chart information, navigation mark distribution, etc. In this paper, laser radar and positioning systems were used in the environmental perception of intelligent SMASS. The decision part includes path planning before sailing and obstacle avoidance during self-service navigation. In this paper, improved PPO algorithms were used for SMASS path planning and obstacle avoidance. The algorithm has the following advantages:

- With autonomous learning ability, the convergence rate was faster than the common calculation method.
- The trained intelligent SMASS navigation system could obtain strong generalization, which would solve different scene problems. For example, it can solve the problem of path planning for SMASS sailing in broad waters, narrow waters, and restricted waters. In local path planning, it could successfully avoid unknown obstacles that do not appear on the electronic chart.
- The path planning problem and SMASS decision problem could be solved simultaneously. SMASS could find the optimal path to the target point through known obstacle information. Under the unknown environment, the SMASS could detect the position of obstacles by laser radar and accurately avoid the obstacles.

### 2.2. Ship Mathematical Model

**Figure 2:**
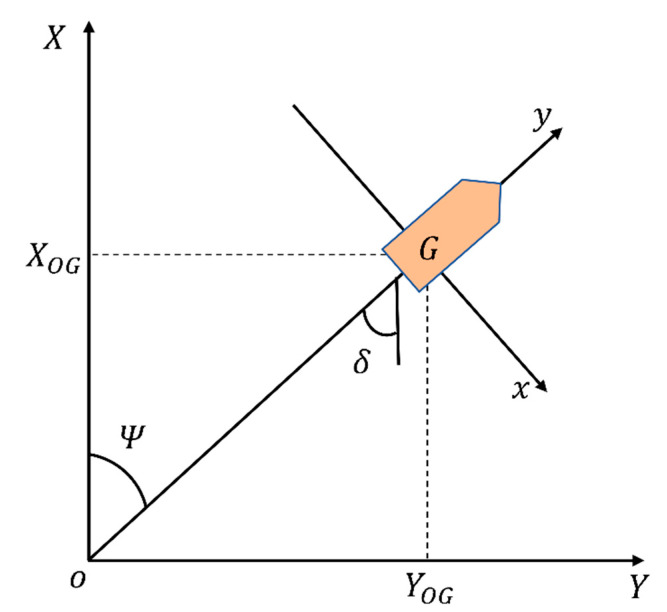
Ship motion mathematical model.

The mathematical model of ship motion is significant for ship motion simulation. The ship motion model can be divided into the linear model and the nonlinear model. The linear model is mainly used to optimize or train the control simulator, neural network decision-making, and controller design [19]. To describe the motion of a ship, a ship motion coordinate system was established, as shown in Figure 2.

In this figure, G represents the position of the center of gravity of the ship, XOY indicates the hydrostatic water plane, O is the origin, $X_{OG}$ represents the projection of the center of ship gravity on the X and Y axes, respectively, ψ is the heading of the ship, and δ indicates the ship rudder angle. Considering only the ship lateral drift velocity v and yaw angular velocity r, the ship motion mathematic model could be expressed as:

(1) $$\left[\begin{array}{l} \dot{v}\\ \dot{r} \end{array}\right]=\left[\begin{array}{l} a_{11}\\ a_{21} \end{array}\right.\left.\begin{array}{l} a_{12}\\ a_{22} \end{array}\right]\left[\begin{array}{l} v\\ r \end{array}\right]+\left[\begin{array}{l} b_{11}\\ b_{21} \end{array}\right]\delta$$

where $a_{11}$, $a_{12}$, $a_{21}$, $a_{22}$, $b_{11}$, and $b_{21}$ are the ship maneuverability parameters [20]. Ignoring the lateral drift velocity v in Equation (1), the response equation of the ship steering rudder to yaw motion can be written as:

(2) $$T_{1}T_{2}\ddot{r}+(T_{1}+T_{2})\dot{r}+r=K_{0}\delta +K_{0}T_{3}\dot{\delta }$$

where $T_{1}$, $T_{2}$, $T_{3}$, and $K_{0}$ are maneuverability indexes. Their values could be estimated by $T_{1}T_{2}=1/(a_{11}a_{22}-a_{12}a_{21})$, $T_{1}+T_{2}=(a_{11}+a_{22})/(a_{12}a_{21}-a_{11}a_{22})$, $T_{3}{=b}_{21}/(a_{21}b_{11}-a_{11}b_{21})$, and $K_{0}=(a_{21}b_{11}-a_{11}b_{21})/(a_{11}a_{22}-a_{12}a_{21})$. Then, the Laplace transform of Equation (2) could be carried out to obtain the transfer function of the ship steering control system, as shown in Equation (3):

(3) $$G(s)=\frac{\psi (s)}{\delta (s)}=\frac{K_{0}(1+T_{3}s)}{s(1+T_{1}s)(1+T_{2}s)}$$

For ships with large inertia, the dynamic characteristics are the most important in the low-frequency range [21]. Thus, let the following formula show that s=jw→0, and, ignoring the second and third-order small quantities, the Nomoto model can be obtained by:

(4) $$G_{\varphi \delta }(s)=\frac{\psi }{\delta }=\frac{K_{0}}{s(T_{0}s+1)}$$

The differential equation form of the Nomoto model is written as shown in Equation (5):

(5) $$T\ddot{\psi }+\dot{\psi }=K\delta$$

The value T represents the coefficient ratio of the inertia moment to the damping moment [22]. A large T value indicates a large inertia moment and a small damping moment during ship motion. The value K actually refers to the angular velocity value of yaw motion by each rudder angle. The large K means a large yaw moment and a small damping moment produced by the rudder.

Taking the ship as a rigid body, when the ship steers at any rudder angle δ, the yaw rate is r. The above formula can be seen as the yaw motion equation of the ship when it steers. When the ship turns, altering her course, at any rudder angle, assuming that the initial conditions are t=0, $\delta =\delta_{0}$, and r=0, the yaw angle at any time can be calculated by KT Equation (6):

(6) $$r=K\delta (1-e^{-t/T})$$

Ship yaw angle r is the derivative of ψ with respect to time. As shown in Equation (7).

(7) $$\psi =K\delta_{0}(t-T+T\cdot e^{-t/T})$$

There are two advantages of using the Nomoto model in this experiment:

- In the low-frequency range, the spectrum of the Nomoto model is very close to that of the high order model.
- The designed controller has low order and is easy to implement.

### 2.3. COLREGs

**Figure 3:**
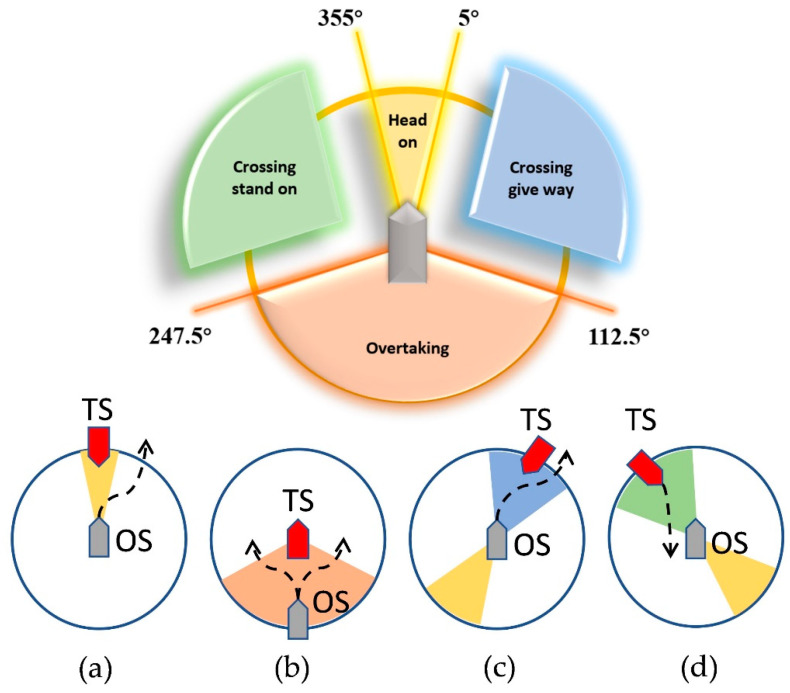
Encounter situations defined by COLREGs: (**a**) Head-on, (**b**) Overtaking, (**c**) Crossing give-way, and (**d**) Crossing stand-on.

To solve SMASS path planning and obstacle avoidance problems based on DRL, maritime collision avoidance rules should be considered. COLREGS is a maritime traffic rule that is stipulated in the high seas and all navigational waters connected to the high seas to ensure the safety of ship navigation, preventing ship collision. Therefore, intelligent ship decision-making systems should also act in accordance with COLREG to ensure the safety of maritime navigation [23]. According to the COLREGS, the relative position of the two ships is divided into four obstacle avoidance strategy regions, such as in Figure 3.

The four collision avoidance rules involved in COLREGS Chapter 2 Regulation 13 to 17 are as follows. The corresponding collision avoidance actions are displayed in Figure 3.

**(1)**  **Head-on**

The encounter situation refers to the opposite or nearly opposite course (where the course usually refers to the bow direction of the ship rather than the track direction) of the two mobile ships under the condition of mutual seeing, and there is a risk of collision. The opposite direction means the relative azimuth of the target ship (TS) and own ship (OS) is in [355°, 360°] or [0°, 5°]. Two ships should alter course port through passing the starboard side of another ship. The head-on situation is displayed in Figure 3a.

**(2)**  **Overtaking**

The overtaking situation means that the speed of the rear ship is greater than that of the front ship. When the own ship chases the target ship in a certain direction 22.5 degrees behind the target ship, the target ship is a stand-on ship, and the own ship should give way to the target ship. The overtaking situation is displayed in Figure 3b.

**(3)**  **Crossing give-way**

When two ships meet and there is a risk of collision, the relative position of the target ship and the own ship is in [5°, 112.5°]. In this case, the own ship should give way to the target ship. According to COLREGs, the own ship should alter her course to starboard to avoid a collision. The crossing give-way situation is displayed in Figure 3c.

**(4)**  **Crossing stand-on**

When two ships meet and the relative position of the target ship and the own ship is in [247.5°, 355°], there is a risk of collision. In this case, the ship is stand-on, and the target ship should give way to the own ship. If the target ship does not take avoidance action timely, the own ship should take action to avoid the collision. The crossing stand-on situation is displayed in Figure 3d.

## 3. Improved PPO Algorithm

### 3.1. Deep Reinforcement Learning

At present, artificial intelligence technologies have developed rapidly; especially after AlphaGo defeated Lee Sedol, the nine-stage chess player, reinforcement learning has risen rapidly to provide new possibilities for intelligent ship path planning. The Q-learning algorithm could obtain the best behavior decision-making through the optimal action-value function [24]. However, the marine environment is too complex, and a ship sailing on the sea faces many uncertainties. The Q-table could seem inadequate in solving complex problems.

The development of deep reinforcement learning is greatly accelerated by neural networks [25]. With the change of the agent’s external environment, through the backpropagation of neural networks, the weights of neural networks could be updated to simulate complex functions. Deep reinforcement learning algorithms are divided into two categories: value learning and strategy learning.

Reinforcement learning based on value function is represented by Deep Q-Learning (DQN), and the problem of correlation and non-static distribution could be solved by the experience replay method. The current Q value is generated by the evaluation network, and the target Q value is generated by the target network [26]. The experience replay stores the transfer samples $\left(s_{t},a_{t},r_{t},s_{t+1}\right)$ from each time step agent that interacts with the environment into the replay memory unit. Then, small-batch data in the memory library are selected for training, but the DQN algorithm is not accurate in estimating the action value Q, so there are some errors. Suppose DQN’s estimate of the real action is unbiased, then the error is noise with an average of 0. $q=\underset{a}{\max}Q(s,a;\omega )$ is maximized based on DQN action a and used to compute $TD_{t\arg et}$. Adding noise to the action-value function will make $q\geq \underset{a}{\max}(S_{t+1},a,\omega )$. Obtaining the Q value at the next moment is an overestimation. Although noise does not change the mean value, it will make the maximum value of Q greater than the maximum value of x. Expectations for the maximum of Q will also be greater than the maximum value of x. Updating DQN estimates at time t with $TD_{t\arg et}$ also means updating itself with itself. Uniform overestimation does not make DQN a problem with action selection because each action overestimation is the same agent and will still choose to score high action. However, non-uniform overestimation will make DQN have problems in the action selection. Double Deep Q-Learning (Double DQN) was proposed by Google DeepMind to solve the overestimation problem of DQN [27]. Although the estimation made by Double Deep Q-Learning is relatively small, its overestimation of the maximum value cannot be solved fundamentally. This is why reinforcement learning based on value learning was abandoned in this paper.

The Actor-Critic (AC) algorithm is representative of strategy learning. There are two neural networks that exist in the AC algorithm. One is used to interact with the environment to select actions, and the other is used to evaluate the quality of actions, and the network parameters are updated by gradient descent. The AC algorithm is good but difficult to converge. Compared with random strategies, deterministic strategies adopt different action probabilities at the same state when solving continuous action problems, but the maximum probability is only one. Double actor neural networks and double critic neural networks were used in the Deep Deterministic Policy Gradient (DDPG) algorithm to improve the convergence of neural networks [28]. The algorithm can only take action with the maximum probability; however, by removing the probability distribution, the algorithm will be much simpler. In 2017, a Proximal Policy Optimization (PPO) algorithm was proposed by OpenAI [29]. The Policy Gradient algorithm is very sensitive to the step size, but it is difficult to select the appropriate step size. If the difference between the new and old strategies is too large, it is not conducive to learning. The problem of uncertain learning rates in the Policy Gradient algorithm could be solved by the PPO algorithm; if the learning rate is too large, then the learned strategy is not easy to converge. On the contrary, if the learning rate is too small, it will take a long time. The proportion of current and previous strategies could be used in the PPO algorithm, which would limit the update range of the current strategy, so that the Policy Gradient algorithm would not be so sensitive to a slightly larger learning rate.

### 3.2. Improved PPO Algorithm

The current and previous strategy networks were used by the traditional PPO algorithm to improve the uncertainty of the learning rate, but they still had a large variance. A generalized advantage estimate was proposed by John Schulman et al. to improve the TRPO algorithm [30], which can also be used to improve the PPO algorithm.

First, the application of baseline in strategy learning should be understood. The baseline could be regarded as a function b independent of action a.

(8) $$\begin{array}{ll} E_{A∼\pi }\left[b\cdot \frac{\partial \ln\pi (A\left|S;\theta \right.)}{\partial \theta }\right] & =b\cdot E_{A∼\pi }\left[\frac{\partial \ln\pi (A\left|S;\theta \right.)}{\partial \theta }\right]\\ & =b\cdot \sum_{a}\pi (a\left|S;\theta \right.)\cdot \frac{\partial \ln\pi (a\left|s;\theta \right.)}{\partial \theta }\\ & =b\cdot \sum_{a}\pi (a\left|s;\theta \right.)\cdot \frac{1}{\pi (a\left|s;\theta \right.)}\cdot \frac{\partial \pi (a\left|s;\theta \right.)}{\partial \theta }\\ & =b\cdot \sum_{a}\frac{\partial \pi (a\left|s;\theta \right.)}{\partial \theta }\\ & =b\cdot \frac{\partial \sum_{a}\pi (a\left|s;\theta \right.)}{\partial \theta }\\ & =0 \end{array}$$

where a is the action taken for the agent, s is the current state, and θ is the network parameter. The essence of the policy function is the probability density function. Taking Equation (9) to the equality of policy gradient update will obtain the advantage function.

(9) $$\begin{array}{ll} \frac{\partial V_{\pi }(S)}{\partial \theta } & =E_{A∼\pi }\left[\frac{\partial \ln\pi (A\left|S;\theta \right.)}{\partial \theta }\cdot Q_{\pi }(S,A)\right]\\ & =E_{A∼\pi }\left[\frac{\partial \ln\pi (A\left|S;\theta \right.)}{\partial \theta }\cdot \left(Q_{\pi }(S,A)-b\right)\right] \end{array}$$

Although the gradient is not affected by the value of b, it affects the Monte Carlo approximation. When b approaches $Q_{\pi }$, the variance of the Monte Carlo approximation will decrease, and the convergence rate will improve. The value of b is $V_{\pi }(S_{t})$, where $V_{\pi }(S_{t})$ is independent of action a, and then the advantage function is obtained. The action value function can be seen as the conditional expectation of the return value $U_{t}$ to $s_{t}$, $a_{t}$, and the state value function can be seen as the conditional expectation of the action value function to $s_{t}$; thus, the equation can be obtained:

(10) $$\begin{array}{ll} Q_{\pi }(s_{t},a_{t}) & =E_{S_{t+1},A_{t+1}}[R_{t}+\gamma Q_{\pi }(s_{t+1},a_{t+1})]\\ & =E_{S_{t+1}}\left[R_{t}+\gamma E_{A_{t+1}}(Q_{\pi }(s_{t+1},a_{t+1}))\right]\\ & =E_{S_{t+1}}[R_{t}+\gamma V_{\pi }(S_{t+1})] \end{array}$$

(11) $$\begin{array}{ll} V_{\pi }(S_{t}) & =E_{A_{t}}[Q_{\pi }(S_{t},A_{t})]\\ & =E_{A_{t}}\left[E_{S_{t+1}}[R_{t}+\gamma V_{\pi }(S_{t+1})]\right]\\ & =E_{A_{t},S_{t+1}}[R_{t}+\gamma V_{\pi }(S_{t+1})] \end{array}$$

At this time, the Monte Carlo approximation of $Q_{\pi }$ and $V_{\pi }$ can be obtained:

(12) $$Q_{\pi }(s_{t},a_{t})\approx r_{t}+\gamma V_{\pi }(s_{t+1})$$

(13) $$V_{\pi }(s_{t})\approx r_{t}+\gamma V_{\pi }(s_{t+1})$$

Because the value b in the dominant function is $V_{\pi }\left(s_{t}\right)$, it is a definite value, so it is not necessary to use the Monte Carlo approximation. The unbiased estimation of the strategy gradient can be expressed as:

(14) $$\begin{array}{ll} g(a_{t}) & =\frac{\partial \ln\pi (a_{t}\left|s_{t};\theta \right.)}{\partial \theta }(Q_{\pi }(s_{t},a_{t})-V_{\pi }(s_{t}))\\ & \approx \frac{\partial \ln\pi (a_{t}\left|s_{t};\theta \right.)}{\partial \theta }\cdot (r_{t}+\gamma V_{\pi }(s_{t+1})-V_{\pi }(s_{t}))\\ & \approx \frac{\partial \ln\pi (a_{t}\left|s_{t};\theta \right.)}{\partial \theta }\cdot (r_{t}+\gamma V_{\pi }(s_{t+1};\omega )-V_{\pi }(s_{t};\omega )) \end{array}$$

We can define $\eta_{t}^{V}=r_{t}+\gamma V_{\pi }(s_{t+1})-V_{\pi }(s_{t})$ and subtract the K−step advantage from the baseline function, then we can obtain the following equation:

(15) $${\hat{G}}_{t}^{\left(\infty \right)}=\sum_{k=0}^{\infty }\gamma^{k}\eta_{t+1}^{V}=-V(s_{t})+\sum_{k=0}^{\infty }\gamma^{k}r_{t+1}$$

Therefore, the generalized advantage estimation can be obtained. The formula is as follows:

(16) $$\begin{array}{ll} {\hat{G}}_{t} & =(1-\lambda )({\hat{G}}_{t}^{\left(1\right)}+\lambda {\hat{G}}_{t}^{\left(2\right)}+\lambda^{2}{\hat{G}}_{t}^{\left(3\right)}+\ldots )\\ & =(1-\lambda )(\eta_{t}^{V}+\lambda (\eta_{t}^{V}+\gamma \eta_{t+1}^{V})+\lambda^{2}(\eta_{t}^{V}+\gamma \eta_{t+1}^{V}+\gamma^{2}\eta_{t+2}^{V})+\ldots )\\ & =(1-\lambda )\left(\eta_{t}^{V}\left(\frac{1}{1-\lambda }\right)+\gamma \eta_{t+1}^{V}\left(\frac{\lambda }{1-\lambda }\right)+\gamma^{2}\eta_{t+1}^{V}\left(\frac{\lambda^{2}}{1-\lambda }\right)+\ldots \right)\\ & =\sum_{k=0}^{\infty }{\left(\gamma \lambda \right)}^{k}\eta_{t+1}^{V} \end{array}$$

The loss function of the PPO algorithm is:

(17) $$L^{PPO}(\theta )=E\left[\min(\mu_{\theta }^{t}G^{t},clip(\mu_{\theta }^{t},1-\varepsilon ,1+\varepsilon ))G^{t}\right]$$

(18) $$\mu_{\theta }^{t}=\frac{\pi (a_{t}\left|s_{t}\right.)}{\pi_{old}(a_{t}\left|s_{t}\right.)}$$

**Figure 4:**
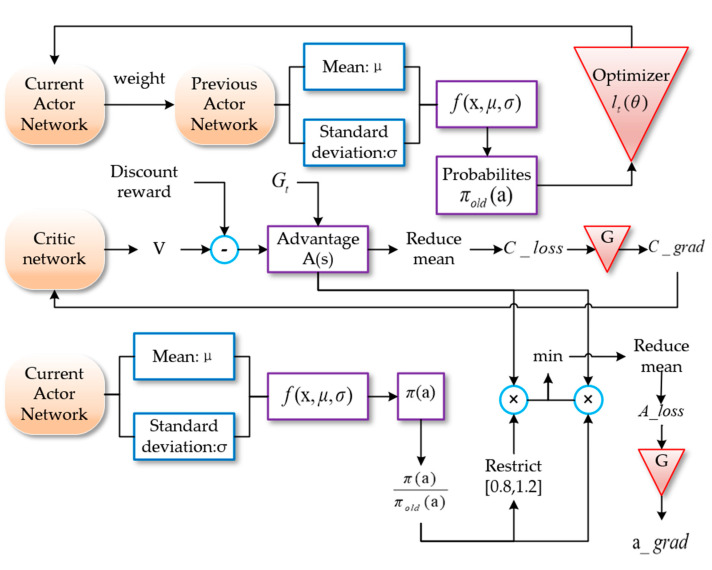
Loss function structure of PPO algorithm.

In this equation, $\mu_{\theta }^{t}$ is the ratio of probability. The ratio of the probability is that the strategy before updating takes a specific operation in a specific state to the probability that the current strategy takes the same operation in the same state. The ratio is between 1−ε and 1+ε according to the range of the super parameter ε. Therefore, there is a great change between the previous strategy and the current strategy. The PPO loss iteration is shown in Figure 4.

## 4. Neural Network Design and Reward Function

### 4.1. Network Construction and Input and Output Information

State information is input by the actor network and critic network in the PPO algorithm. Two-dimensional plane coordinates of the ship ($x_{p}$, $y_{p}$); rudder angle and rudder angular velocity of the operating system (δ, $\delta_{1}$); and 24 laser radar vector lines $\left(\chi_{1},\chi_{2},\chi_{3}\ldots \chi_{24}\right)$ were used as the state information of the environment.

**Figure 5:**
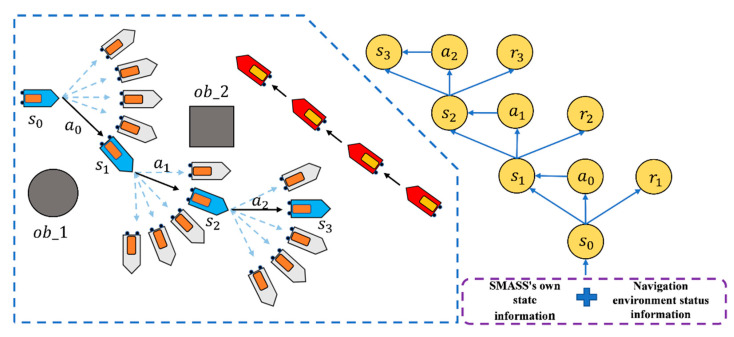
The update process of the SMASS collision avoidance algorithm.

To avoid collisions with other ships, the navigator should adjust the direction of their own ship to ensure the navigation safety of ships in designated waters. The collision avoidance method of an autonomous ship can be created through a sufficient learning process by simulating the appropriate decision-making skills that the navigator could acquire over a long period of experience [31]. In this experiment, the output data are the rudder angle of the SMASS. The course and path of the SMASS would be affected by the change of rudder angle. The altering course to port is defined as negative, and altering course to starboard is defined as positive. The action space of this experiment is [−45°, −25°, 0°, 25°, 45°]. The obstacle avoidance process of the SMASS is shown in Figure 5.

**Figure 6:**
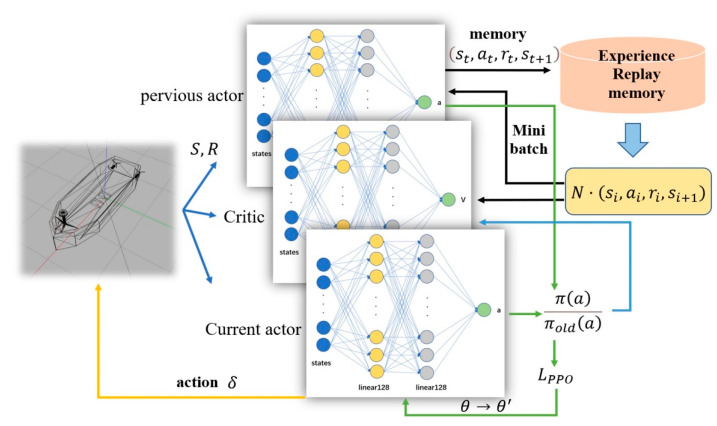
Flow chart of the PPO algorithm and environment interaction. Input is state vector S, output is ship steering angle δ. Both critic network and actor network are connected by a linear layer with 128 neurons using the Relu activation function.

In this paper, the deep neural network was used to fit the policy function *π*. Among them, the actor network adopted a two-layer full connection layer with 128 neurons. The Relu activation function was used and the network input was state S. The obtained expectation and standard deviation were put into the Gaussian distribution, the probability density function was obtained using the strategy distribution, and the probability corresponding to different action a was the output. The critic network adopted two fully connected layers with 128 neurons and the Relu activation function. The network input was state S, the output of the actor selected action score. The PPO algorithm and environment interaction process are shown in Figure 6.

The probability obtained by the previous strategy was optimized with other relevant parameters, and the difference in the new_Actor network was obtained. The obtained difference was put into the new_Actor, so that the strategy of the global network is new, and the strategy of the regional network is old. The critic network output is the value V, using discount reward, value subtraction, and generalized advantage estimate optimization to obtain the advantage function. Then, the gradient descent algorithm was used to calculate the error and update the network parameters. The proportion of the current and previous strategies was multiplied by the advantage function. One part was directly multiplied, and the other part was multiplied after 1−ε and 1+ε, according to the range of the super parameter ε. The minimum value of the two was taken, and then the error was calculated.

To break the correlation of data and ensure the convergence of policy functions, an empirical playback memory can be set to store the historical motion state. Under each time step t, the intelligent ship entered a new state after interacting with the environment, and the updated state was put into the memory. In the process of the neural network training, a small batch of state samples were extracted from the memory to ensure the stability of the training.

### 4.2. Reward Function

**Figure 7:**
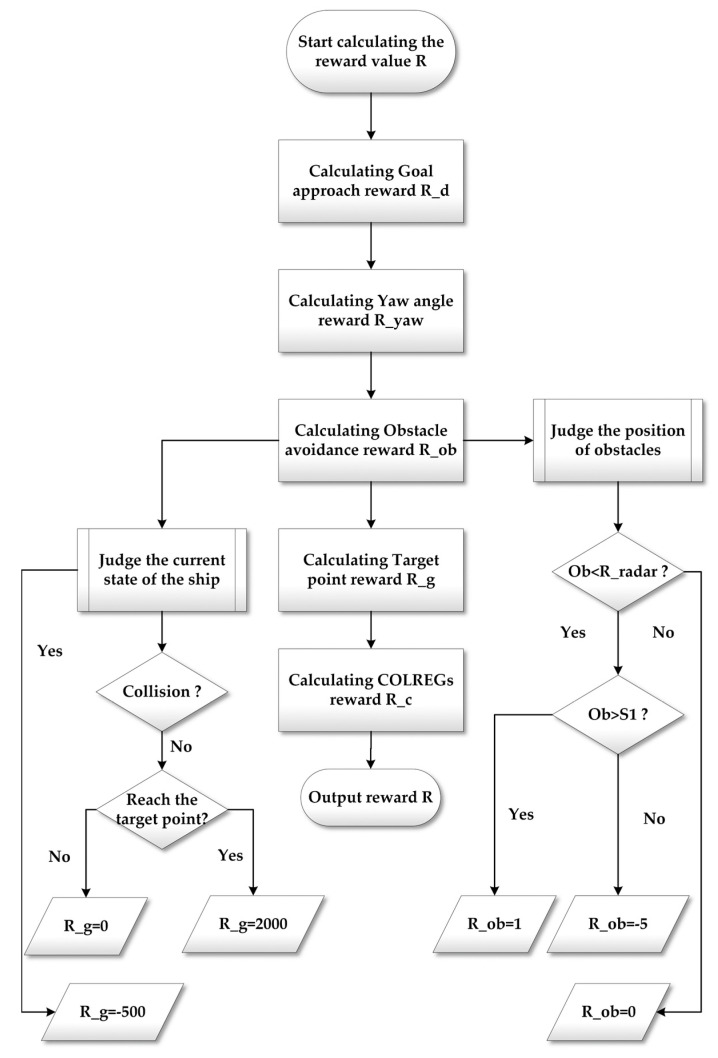
Calculation process of the reward function.

According to the task of SMASS path planning and obstacle avoidance, the reward function was set to the following five parts: goal approach reward, yaw angle reward, target point reward, obstacle avoidance reward, and COLREGs reward as shown in the Figure 7.

**(1)**  **Goal approach reward**

The primary task to solve the intelligent SMASS path planning was to make the SMASS reach the target position. The goal approach reward value was set as follows:

(19) $$R\_d=-\lambda_{g}\cdot \sqrt{{\left(x_{p}-x_{g}\right)}^{2}+{\left(y_{p}-y_{g}\right)}^{2}}$$

where $x_{p}$ and $y_{p}$ are the coordinates of the current position of the ship, $x_{g}$ and $y_{g}$ are the coordinates of the target point, and $\lambda_{g}$ is the weight of the target proximity reward.

**(2)**  **Yaw angle reward**

**Figure 8:**
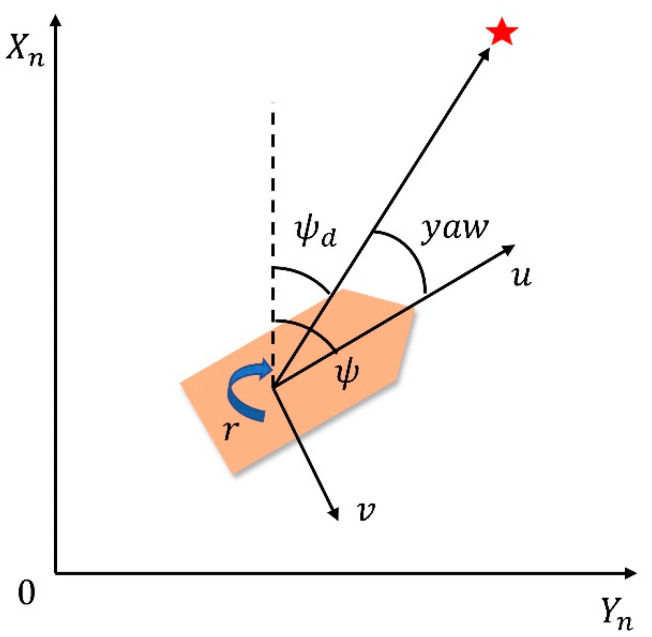
Definition of heading angle error.

When the SMASS is planning the path, the heading angle should be taken as an important indicator. As shown in Figure 8, the connection between the current position of the ship and the position of the target point should be regarded as the shortest distance, and the SMASS motion direction should be along this direction as far as possible. The Yaw angle reward function is set as follows:

(20) $$R\_yaw=tr\cdot \lambda_{a}\cdot 2^{{\left(\varepsilon yaw\right)}^{2}\sqrt{{\left(x_{p}-x_{g}\right)}^{2}+{\left(y_{p}-y_{g}\right)}^{2}}}$$

where yaw is the yaw angle between the SMASS and the target point; tr is the reward coefficient of the yaw angle, which indicates that the reward values obtained from different angles are different; $\lambda_{a}$ is the weight of the yaw angle reward; and ε is the adjustment parameter of the reward value and distance.

**(3)**  **Target point reward**

In order to get the SMASS to the target point, it is necessary to set a reward at the target point position. At the same time, the SMASS should also receive a negative reward when it collides with obstacles during navigation. The reward value is set as follows:

(21) $$R\_g=\left\{\begin{array}{l} -500\;collision\\ 2000\;goal \end{array}\right.$$

**(4)**  **Obstacle avoidance reward**

**Figure 9:**
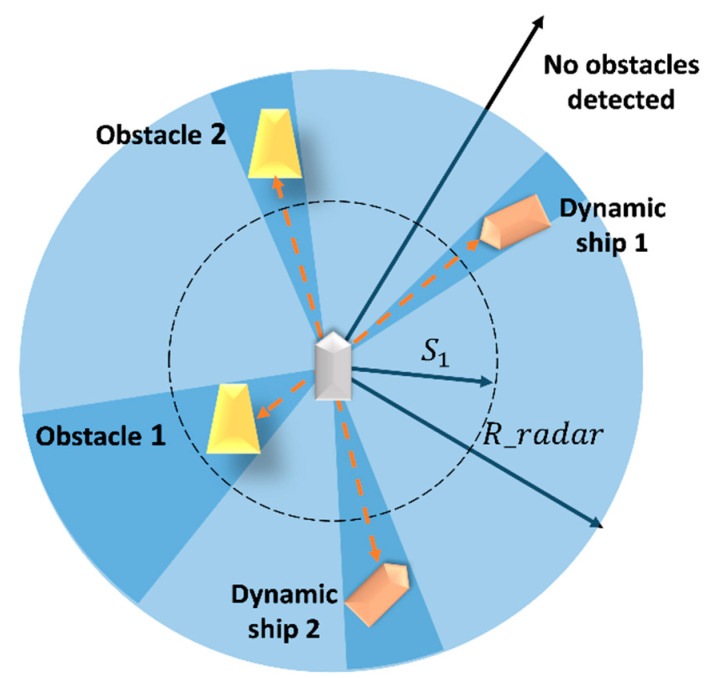
The process of obstacle detection by SMASS laser radar.

The laser radar detection range of the SMASS is a circle, launching 24 detection lines from the center of the circle; R_radar is the radar radius, and the reward is 0 when the static obstacle is outside the radar radius. As shown in Figure 9, $S_{1}$ is set as the safe distance between the SMASS and the obstacle. When the distance between the SMASS and the obstacle is less than $S_{1}$, a negative reward will be obtained. The reward value is set as follows:

(22) $$R\_ob=\left\{\begin{array}{l} 0\;ob>R\_radar\\ -5\;ob<R\_radar,ob<S_{1}\\ 1\;ob<R\_radar,ob>S_{1} \end{array}\right.$$

**(5)**  **COLREGs reward**

In order to make the trained SMASS behavior satisfy COLREGs, a COLREGs reward function was introduced. The distance between SMASS and the target point was designed in the COLREGs reward.

While SMASS needs to keep heading, the rudder angle should be 0. In addition, when SMASS needs to avoid obstacles or target ships, she should alter her course to starboard. These are defined as satisfying COLREGs. Otherwise, SMASS should alter her course to port or hold heading after encountering obstacles or target ships, which is considered to be a violation of COLREGs. When the SMASS operations comply with COLREGs, the SMASS would obtain positive rewards. However, when SMASS violates COLREGs, it will be punished. Hence, the reward function can be set as follows:

(23) $$R\_c=\left\{\begin{array}{l} 0\;\;contrary\;to\;COLREGs,\\ \lambda_{c}\cdot \sqrt{{\left(x_{p}-x_{g}\right)}^{2}+{\left(y_{p}-y_{g}\right)}^{2}}\;else. \end{array}\right.$$

where $\lambda_{c}$ is the weight of the COLREGs reward function.

Therefore, the calculation process of the total reward function is shown in Figure 7 and is expressed as follows:

(24) R=R_d+R_yaw+R_g+R_ob+R_c

## 5. Simulation

### 5.1. Design of Simulation

**Figure 10:**
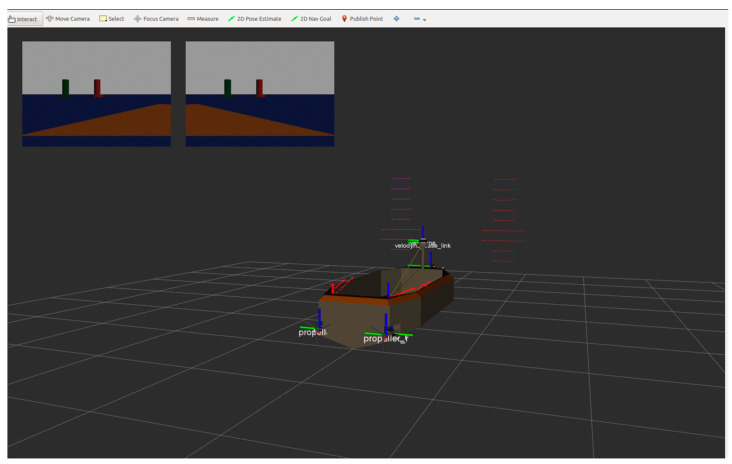
Ship model built in Gazebo simulation environment.

The training environment is necessary for the intelligent SMASS deep reinforcement learning. A designed unmanned ship training environment can quickly test algorithms [32]. Hence, multiple simulation scenarios were set up to train mobile SMASS for path planning. Based on the improved PPO algorithm proposed above and the construction of the neural network framework, the neural network was trained. The computer configuration was as follows: Intel Core i9-11900K, NVIDIA GTX3090, 24 G video memory, 32 G main memory, and 512 G SSD storage. Gazebo and VScode were used for joint simulation and established a three-dimensional navigation environment in Gazebo to simulate different waters and build a SMASS model, as shown in Figure 10.

Some restrictions were attached to the SMASS model. SMASS cannot slow down her speed and can only alert her course during the voyage. The SMASS inertia was appropriately increased to simulate the real motion state of the SMASS. In the SMASS steering phase, with the increase of the rudder angle, the rudder transverse force and rudder force turn the SMASS moment. In the transition stage, the transverse velocity and angular velocity were generated under the action of transverse force and rudder force transfer torque, and the increasingly obvious oblique shipping motion made the ship enter the accelerated rotation state. When the SMASS moved in a fixed-length cycle, the steering force transfer torque, drift angle hydrodynamic transfer torque, and resistance transfer torque were balanced. The acceleration of the rotational angular is zero, and the rotational angular velocity was the largest and most stable at this value. This experiment assumed that the SMASS navigated in still water.

### 5.2. Network Training Process

Experimental parameter settings are shown in Table 1. The Gazebo environment platform module is responsible for generating a navigation environment and simulating SMASS simulation. The environment module could generate and calculate SMASS position and SMASS movement information. When the SMASS reached the target, the training task was over, and entered the next training. When the SMASS encountered obstacles, it stopped training immediately and was placed in the initial position for the next training. The SMASS obstacle avoidance decision training process was divided into two environments, environment one (Env1) and environment two (Env2).

**Figure 11:**
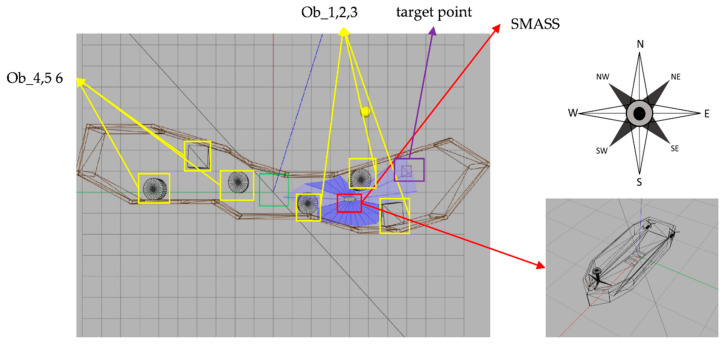
Gazebo simulation environment (Env1). From right to left are six obstacles (ob1, ob2, ob3, ob4, 0b5, and ob6), the blue part is the laser radar range, and the blue line is the laser radar detection line. The left purple box is the target point.

In the experiment, the initial position of the SMASS in the simulation environment was (0,0). There were six static obstacles in the simulation environment, and the coordinates of these six static obstacles were (0.46, 1.78), (−0.57, −1.75), (1.68, 3.78), (0.62, −4.44), (0.13, 6.08), and (−1.15, −6.18). There were two target points, and the coordinates were (1.00, −7.00) and (2.00, 7.00), as shown in Figure 11. In the early stage of environmental interaction, ships extremely lacked driving experience and collision avoidance experience. The trained SMASS could not navigate towards the target and avoid a collision.

**Figure 12:**
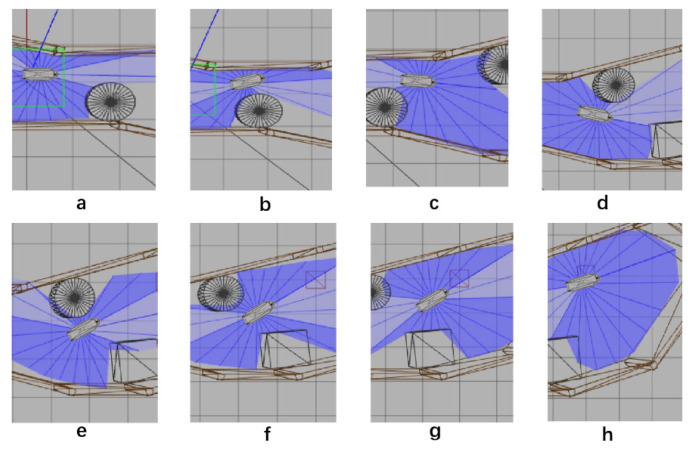
The processes of SMASS avoiding obstacle in Env1 after 1200 training times. Subfigures (**a**–**c**) show the process of SMASS avoiding ob3. Subfigures (**d**–**f**) show the process of SMASS avoiding ob2. Subfigures (**g**,**h**) show the process of SMASS avoiding ob1.

After 1000 training times, SMASS could avoid obstacle 1 and obstacle 2. When the SMASS sailed on the port side of obstacle 2, the course remained unchanged. When encountering obstacle 2, the SMASS took two consecutive port alters of 25° and moved towards the upper right under obstacle 2. When it was 0.6 miles from obstacle 1, her course to port was altered to 45°, along with obstacle 1 upward obliquely. The SMASS continuously steered port and starboard and changed course during movement, but the SMASS could not reach the target point and collided with the environmental framework during the wandering process. SMASS collision avoidance obstacles 1, 2, and 3 are shown in Figure 12.

After training about 1200 times, the SMASS successfully reached the first target point. Subsequently, the SMASS continuously altered her course to port 45° and sailed to the target point 2. When the SMASS passed under obstacle 3 and navigated towards obstacle 4, her course was altered to starboard 25°, then port and starboard rudder were altered continuously to ensure heading stability.

**Figure 13:**
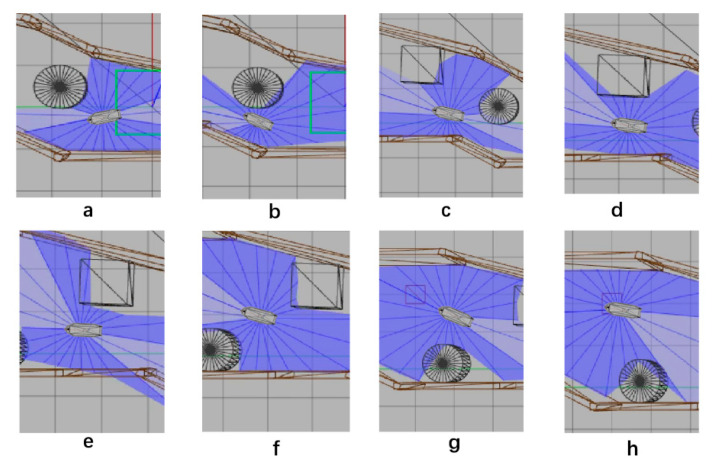
The processes of SMASS avoiding obstacle in Env1 after 1500 training times. Subfigures (**a**–**c**) show the process of SMASS avoiding ob4. Subfigures (**d**–**f**) show the process of SMASS avoiding ob5. Subfigures (**g**,**h**) show the process of SMASS avoiding ob6.

**Figure 14:**
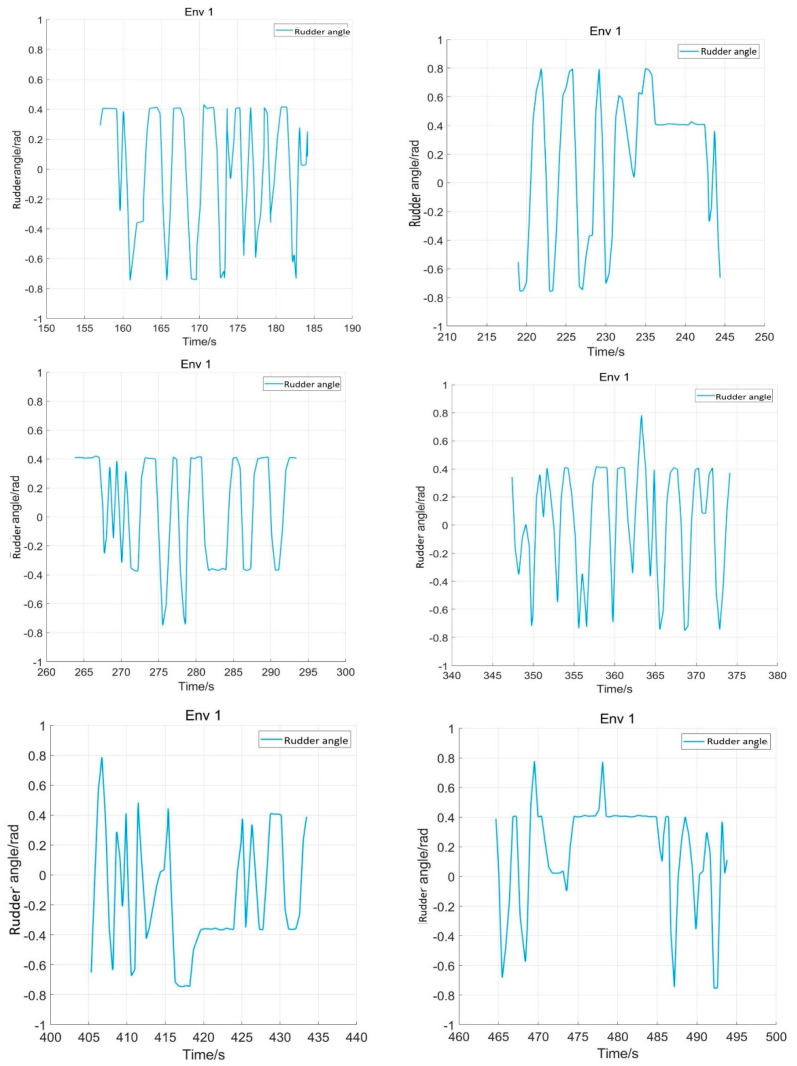
Rudder angle changes of the SMASS sailing in environment 1.

After training 1500 times, the SMASS could maintain her course and sail to the target point. The SMASS first altered her course to port 25° close to the upper starboard of obstacle 6, and then turned starboard by 25° twice in succession, passing over Obstacle 6, successfully reaching the target point 2. The collision avoidance process is shown in Figure 13. In training environment one, the SMASS successfully avoided six obstacles. In the process of SMASS obstacle avoidance, the change curve of the SMASS steering angle with time is shown in Figure 14.

**Figure 15:**
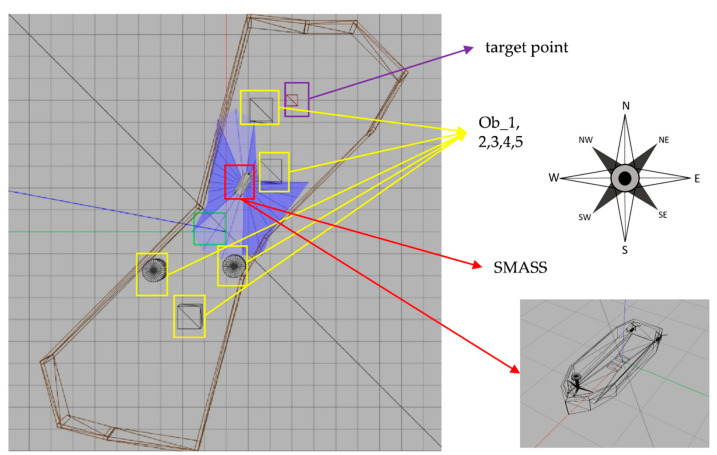
Gazebo simulation environment (Env2). From up to down, there are five obstacles (ob1, ob2, ob3, ob4, and ob5); the blue part is the laser radar range, and the blue line is the laser radar detection line. The purple box is the target point.

There were five obstacles in the second simulation environment, and the coordinates of these five obstacles were (−1.7, 3.2), (−1.6, −0.5), (2.7, −2.0), (5.4, −1.6), and (−3.8, 1.6). The coordinates of the two target points were (6.0, −3.0) and (−4.5, 3.0), as shown in Figure 15.

**Figure 16:**
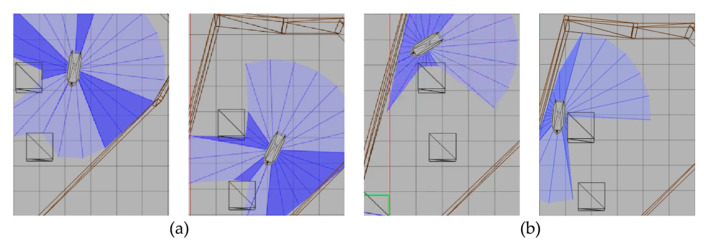
SMASS avoids Obstacle 1 in Env 2. Figure (**a**) shows the obstacle avoidance process of the ship after 1400 training times. Figure (**b**) shows the obstacle avoidance process of the ship after 1200 training times.

After training about 1200 times, the SMASS frequently operated the rudder and reached the first target point. In the process of sailing to the second target point, the SMASS chose to sail around obstacle 1 from above, as shown in Figure 16b. After reaching the target point, the SMASS chose to alter course to port 45° to sail a distance on the left upper side, and then frequently operated the rudder. When the SMASS reached the top left of obstacle 1, the SMASS chose to alter the course to port 45° to drive down.

**Figure 17:**
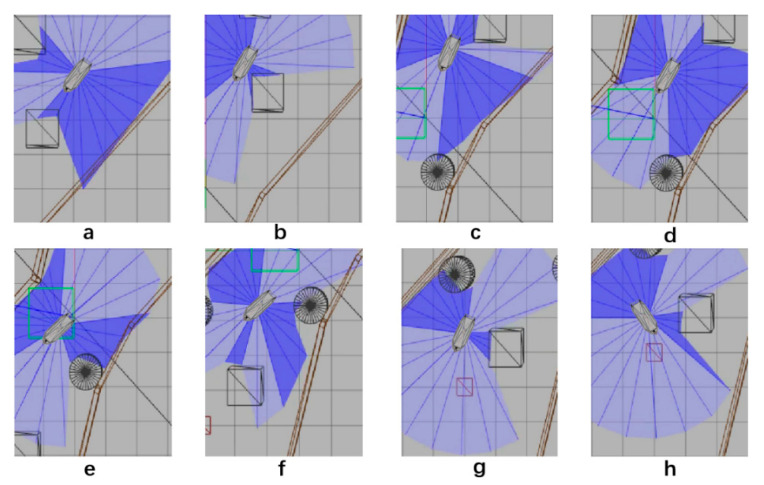
The processes of SMASS avoiding obstacle in Env2. Subfigures (**a**–**c**) show the process of SMASS avoiding ob1 and ob2. Subfigures (**d**–**f**) show the process of SMASS avoiding ob3. Subfigures (**g**,**h**) show the process of SMASS avoiding ob4 and ob5.

**Figure 18:**
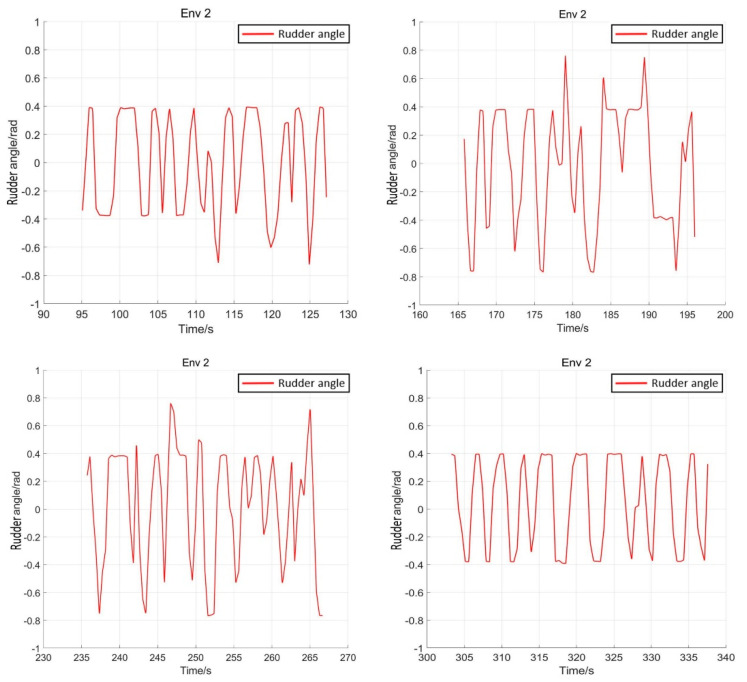
Rudder angle changes of the SMASS sailing in environment 2.

After training 1400 times, the SMASS almost did not collide with five obstacles or enter the minimum distance $S_{1}$ between the SMASS and the static obstacle. The reward value obtained by the SMASS crossing between obstacle 1 and obstacle 2 was greater than that obtained by the SMASS bypassing above obstacle 1. As shown in Figure 16a, when the distance between the SMASS and obstacle 1 was greater than 0.5 miles, the SMASS altered her course to starboard 25°. When the SMASS was 0.4 miles away from obstacle 2, the SMASS chose to alter her course to starboard 25° and moved forward 0.5 miles. Subsequently, the SMASS altered her course to port and avoided obstacle 2. At the same time, when the SMASS arrived at target 2 and got ready to return to target 1, the reward value obtained by the SMASS passing through the left side of obstacle 5 was larger than that passing through the right side. After passing obstacle 5, the SMASS chose to alter her course to port by 25°. The SMASS altered her course to starboard 45° after passing through obstacle 5. The process of SMASS obstacle avoidance is shown in Figure 17. In the process of SMASS obstacle avoidance, the change of the SMASS steering angle is shown in Figure 18.

### 5.3. Comparison Experiment

**Figure 19:**
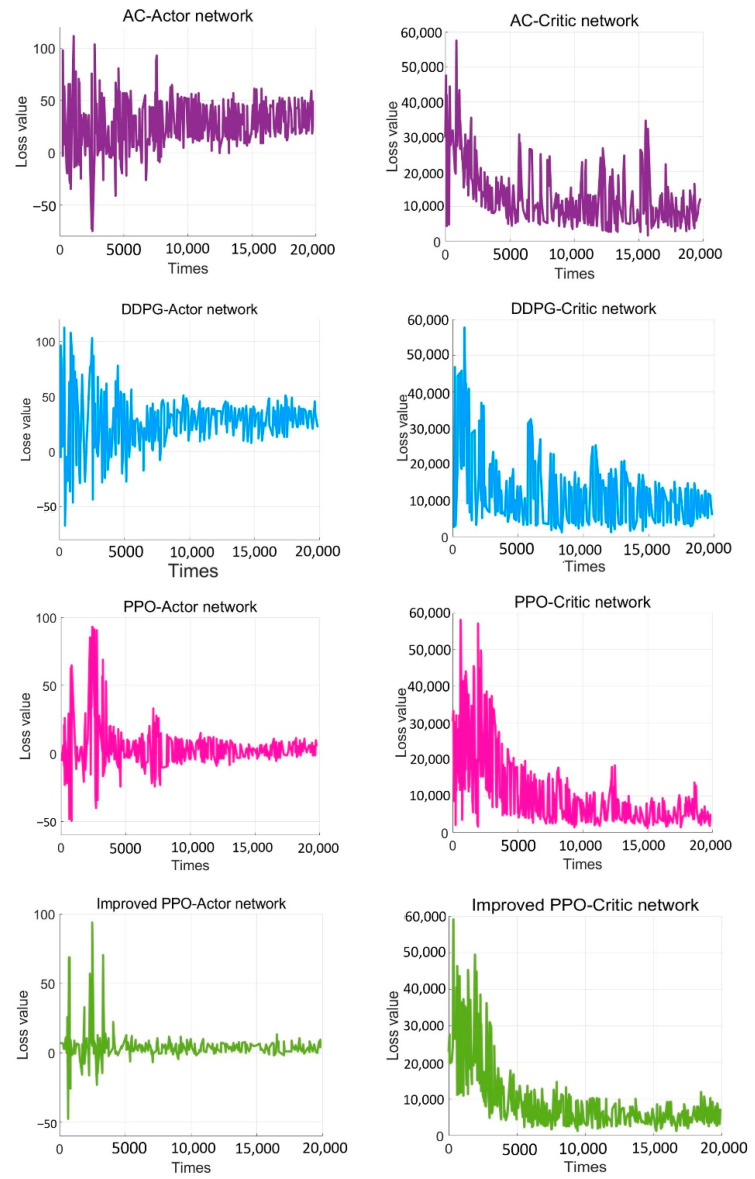
The comparative experiment of the convergence curve.

To verify the effectiveness of the improved PPO algorithm, this paper compared the improved PPO algorithm with the other classic strategy-based reinforcement learning algorithms (such as the AC algorithm, DDPG algorithm, and traditional PPO algorithm). As shown in Figure 19, after training 20,000 times, the actor-network in the AC algorithm converged after training 11,000 times, and the critic network converged after training 10,000 times. The results showed that the convergence rate of the AC algorithm was not satisfied, and the loss value was high. While the DDPG algorithm converged after about training 10,000 times, the algorithm still had the problem of high loss value. When solving SMASS decision-making problems, the traditional PPO algorithm converged after 8000 training times, which was better than the AC algorithm and DDPG algorithm. However, the improved PPO algorithm converged after 6000 training times; the convergence rate was significantly better than the traditional PPO algorithm, and the loss was greatly improved. Hence, it can be found that the convergence rate of the improved PPO algorithm could increase by about 25% compared to the traditional PPO algorithm. Compared with the traditional DDPG and AC algorithms, the convergence rate of the improved PPO algorithm could increase by about 50%.

The Generalized Advantage Estimation Algorithm directly affects the convergence speed and convergence quality of the PPO algorithm. In this experiment, four groups of comparison experiments were conducted to prove the influence of differences in the generalized advantage estimation on the PPO algorithm. Taking the training environment as an example, four λ values were selected for comparative experiments, which were 0.8, 0.9, 0.95, and 0.99, respectively.

**Figure 20:**
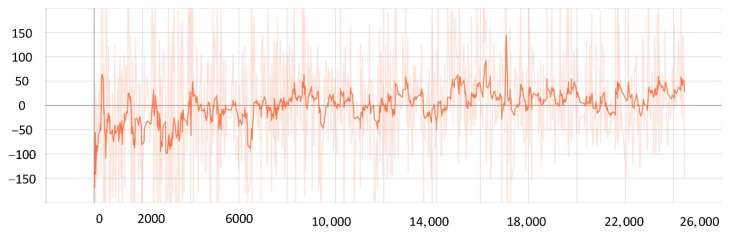
The loss function value change with episodes of actor network when λ was 0.8.

**Figure 21:**
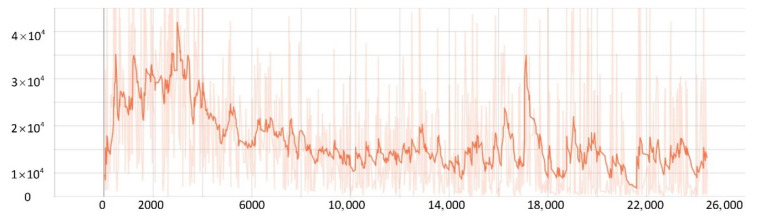
The loss function value change with episodes of critic network when λ was 0.8.

The convergence of actor and critic networks when λ was 0.8 is shown in Figure 20 and Figure 21. The convergence of the actor network was not obvious, and the critic network was not converged obviously after 24,000 training sessions.

**Figure 22:**
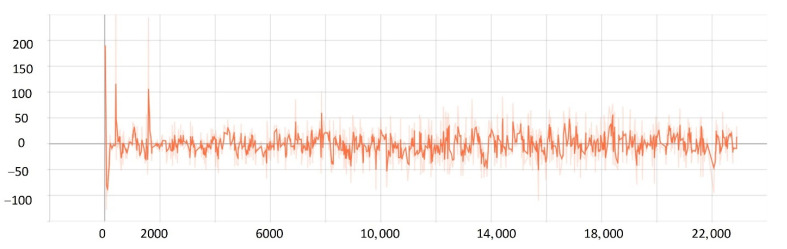
The loss function value change with episodes of actor network when λ was 0.9.

**Figure 23:**
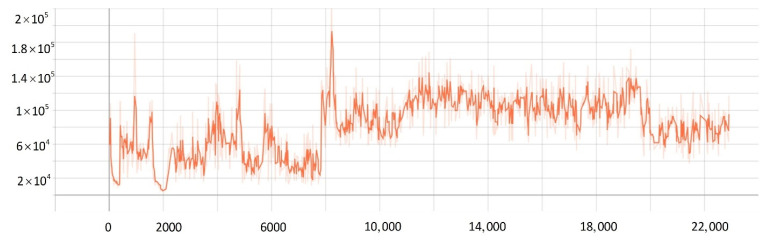
The loss function value change with episodes of critic network when λ was 0.9.

The convergence of actor and critic networks when λ was 0.9 is shown in Figure 22 and Figure 23. Compared with the actor network convergence curve when λ was 0.8, the actor network convergence was better, but the critic network still did not converge after 22,000 training sessions.

**Figure 24:**
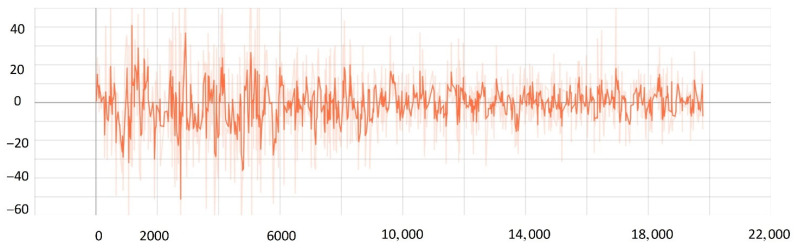
The loss function value change with episodes of actor network when λ was 0.95.

**Figure 25:**
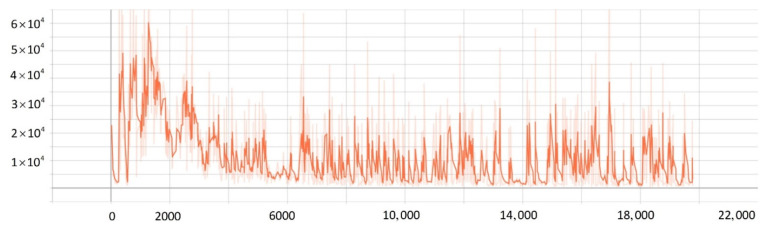
The loss function value change with episodes of critic network when λ was 0.95.

The convergence of actor and critic networks when λ was 0.95 is shown in Figure 24 and Figure 25. The convergence rate of the actor network was faster than when λ was 0.9 in the early convergence effect, and the overall convergence trend was shown. In addition, the convergence effect of the critic network was significantly better than when λ was 0.9.

**Figure 26:**
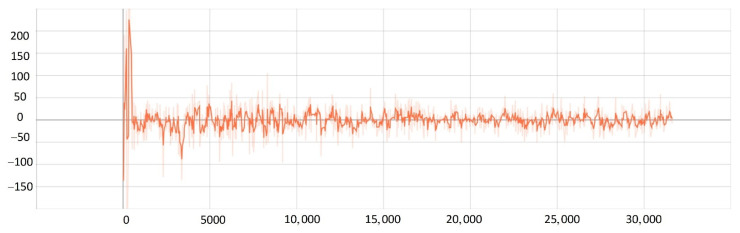
The loss function value change with episodes of actor network when λ was 0.99.

**Figure 27:**
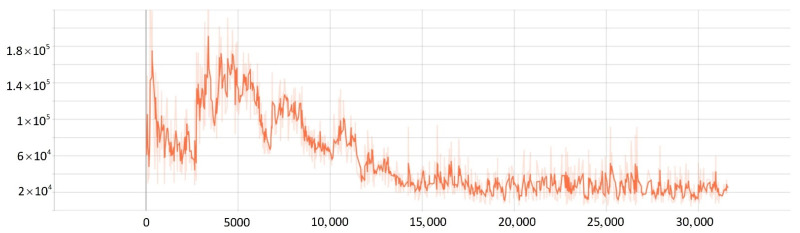
The loss function value change with episodes of critic network when λ was 0.99.

The convergence of actor and critic networks when λ was 0.99 is shown in Figure 26 and Figure 27. The convergence rate of the actor network was much faster than that of the curve when λ was 0.95. In addition, when λ was 0.99, the convergence quality and stability of the actor network and critic network were better than the curve when λ was 0.95.

### 5.4. Verification Simulation

Generalizability refers to the ability of trained models to apply to new data and make accurate predictions. When the training is insufficient, the fitting ability of the decision-making system is not obvious. The disturbance of training data is insufficient to make the decision-making system change significantly. With the increase of training times, the fitting ability of the decision-making system is gradually enhanced. The disturbance can be detected by the decision-making system. A model is often trained too well on training data, that is, overfitting, so that it cannot be generalized. In order to prove the generalization of the proposed SMASS intelligent obstacle avoidance model in this paper, several different simulation environments were constructed to verify the generalizability of the trained SMASS obstacle avoidance network.

**Figure 28:**
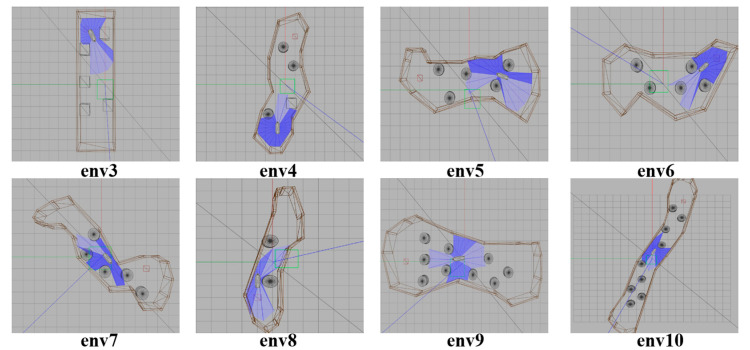
Experimental environment to verify the universality of the SMASS decision-making network.

**Figure 29:**
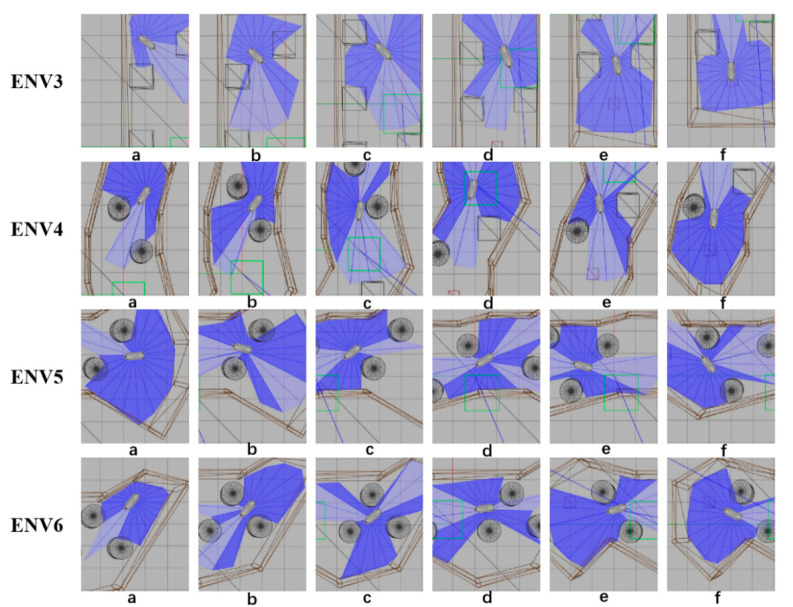
Obstacle avoidance process from environment 3 to environment 6. The SMASS collision avoidance process of each environment is shown by six subgraphs (**a**)–(**f**).

**Figure 30:**
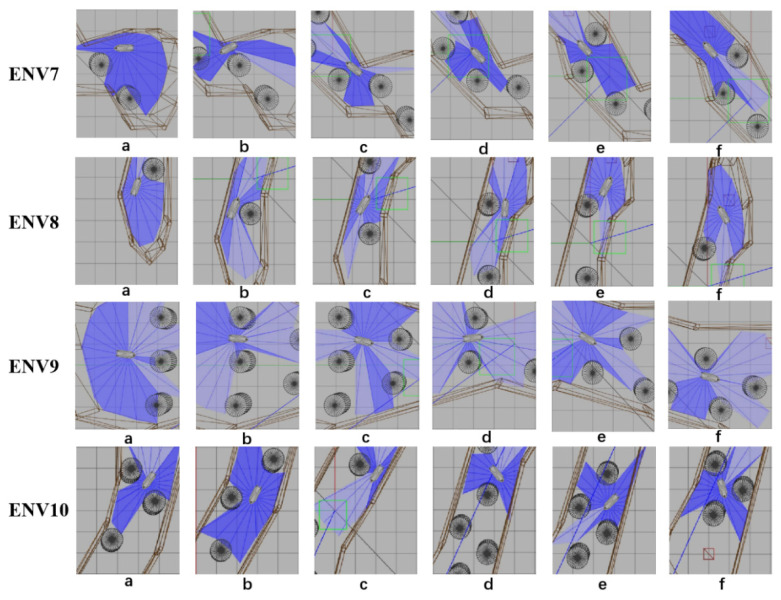
Obstacle avoidance process from environment 7 to environment 10. The SMASS collision avoidance process of each environment is shown by six subgraphs (**a**)–(**f**).

**Figure 31:**
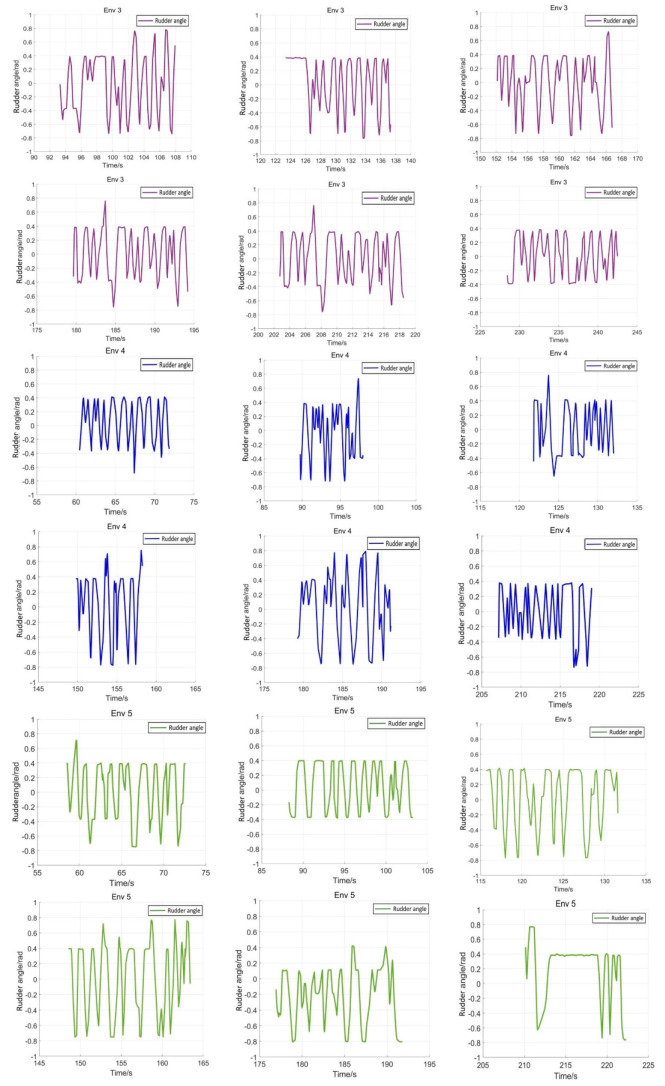
Rudder angle changes of the SMASS sailing in environment 3, environment 4, and environment 5.

**Figure 32:**
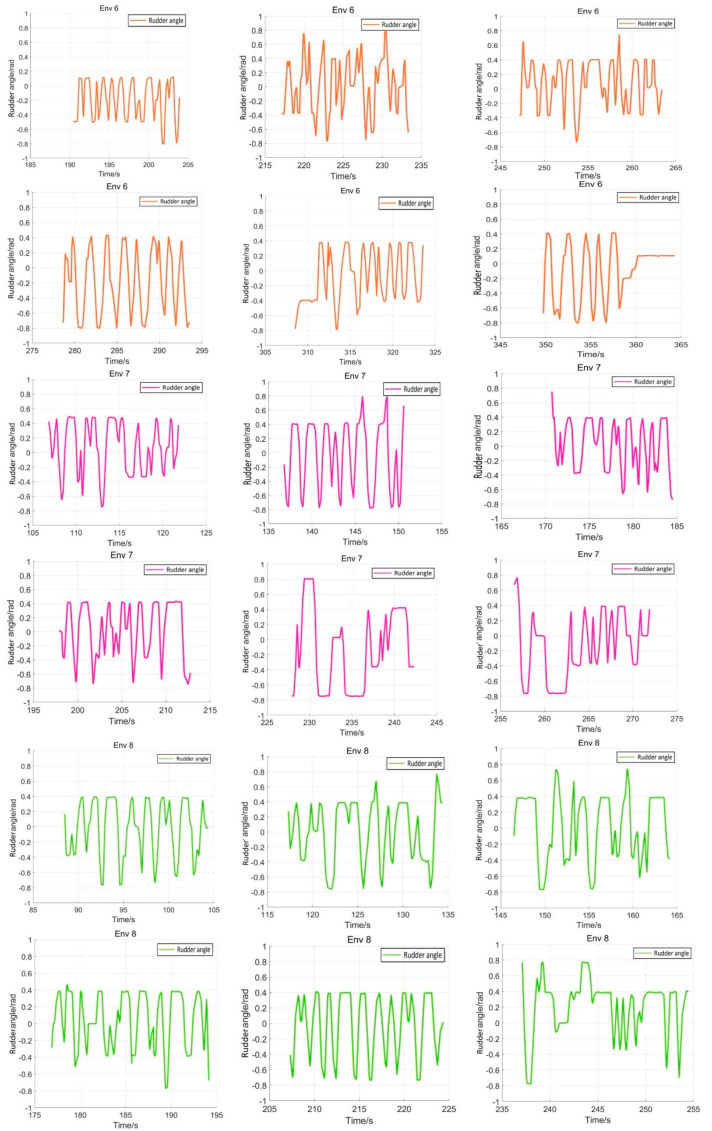
Rudder angle changes of the SMASS sailing in environment 6, environment 7, and environment 8.

**Figure 33:**
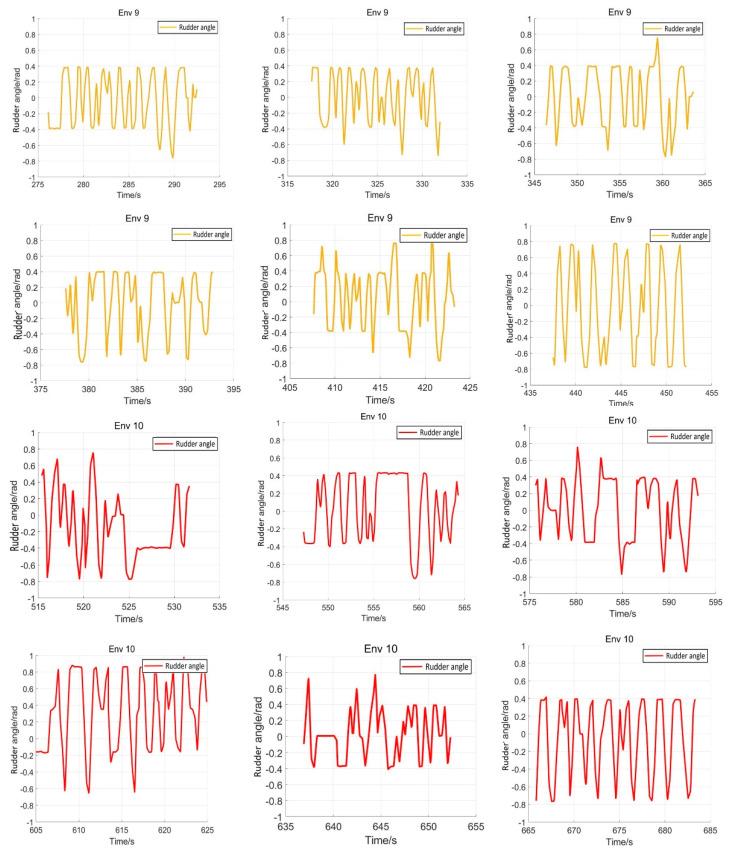
Rudder angle changes of the SMASS sailing in environment 9 and environment 10.

The eight representative simulation environments were extracted and displayed as shown in Figure 28. The initial and end positions of each environment were shown in Table 2. There were five obstacles in environment 3. Environments 4, 5, and 6 were used to simulate the navigation of SMASS in relatively narrow waters. The number of obstacles in environment 7 was not too much, but the environment was more complex. There were only two obstacles in environment 8, but the navigable waters were very narrow to simulate the SMASS obstacle avoidance in narrow waters. Environment 9 was relatively open, but there were multiple obstacles located along a line. The environment was used to test whether the SMASS could find the optimal path when there were multiple obstacles in the environment. In environment 10, the navigation area with more obstacles was very narrow, which could be used to simulate the SMASS complex obstacle avoidance navigation in complex narrow waters. In each environment, the collision avoidance processes from the starting position to the end position were described by six graphs (as shown in Figure 29 and Figure 30). Moreover, the SMASS steering rudder angle of collision avoidance processes in each environment are shown in Figure 31, Figure 32 and Figure 33.

In addition, the avoidance simulations of sailing target ships were carried out to verify the trained SMASS obstacle avoidance capability. Taking the No. 9 environment as an example, these sailing target ships met the trained SMASS under the different collision encounter situations, and the trained SMASS could avoid them accurately and safely according to COLREGs.

**Figure 34:**
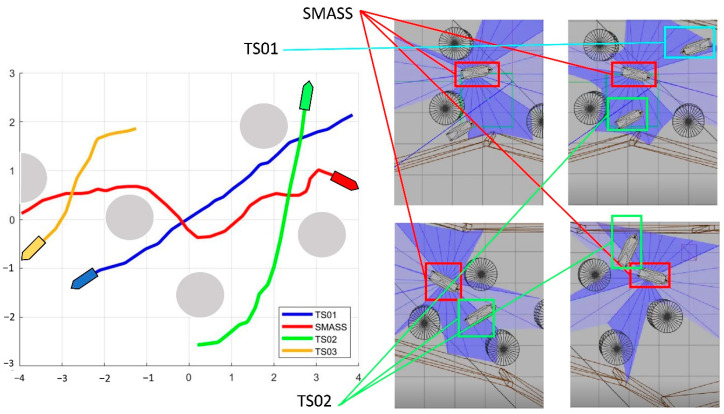
Trained SMASS avoids other ships in environment 9.

As shown in Figure 34, the left side of the figure is the sailing path of the SMASS and three target ships, and the right side is the SMASS avoidance process in the simulation environment. The first target ship (TS01) and the SMASS formed a crossing give-way situation, and the SMASS altered her course to starboard to avoid the first target ship. When the SMASS met the second target ship (TS02), the two ships are formed a crossing stand-on situation. Then, the SMASS kept her course and altered starboard to avoid the second target ship. When the SMASS passed through the middle position, the third target ship (TS03) and the SMASS formed the head-on situation. Then, the SMASS altered course to starboard to avoid the third target ship.

## 6. Conclusions

An improved PPO algorithm for path planning and obstacle avoidance in different complex waters was presented in this paper. SMASS can perform complex local path planning and obstacle avoidance operations when external information is not fully accepted. In this experiment, five factors were considered in the design of the reward function, namely, the relationship between target position, angle, and distance, COLREGs, the reward for safety obstacle avoidance, and whether to reach the target point. This algorithm also performed well in complex waters composed of different numbers of obstacles. The contributions of this experiment are as follows:

- The improved PPO algorithm is superior to other traditional model-free reinforcement learning algorithms based on strategy learning in solving ship decision-making and local path planning problems. The improved PPO algorithm has the advantages of fast convergence and low loss value.
- The improved PPO algorithm has a strong self-learning ability and strong generalization, which could be used to solve the SMASS local path planning and collision avoidance decision-making simultaneously in different complex navigation environments.

Some works should be explored in the future. In the experiment, there are some limitations in setting obstacles into cylinders and squares. Actual obstacles such as islands and navigable areas are not suitable to be set into base shapes. The design of complex obstacles is one of the directions in the future study. In addition, the rudder angle output in this study was the command rudder angle, which has a certain deviation from the execution rudder angle. This is also an important factor to be considered in future studies.

## Figures and Tables

**Table 1:** Experiment parameter information table.

Experimental Parameters	Symbol	Value
Discounted rate	γ	0.95
Lambda	λ	0.99
Clipping hyperparameter	ε	0.20
Target reward weight	$\lambda_{g}$	10.0
Reward coefficient	tr	1.00
Yaw angle reward weight	$\lambda_{a}$	0.30
COLREGs reward weight	$\lambda_{c}$	1.20
Safe distance	$S_{1}$	0.50
Radar radius	R_radar	4.50

**Table 2:** Training environment and verification environment target point coordinates.

Gazebo Environment	Initial Position	End Position
Env 1(Train)	(1.0, −7.0)	(2.0, 7.0)
Env 2(Train)	(6.0, −3.0)	(−4.5, 3.0)
Env 3(Verification)	(5.0, 2.0)	(−4.0, 1.0)
Env 4(Verification)	(5.0, −1.0)	(−5.0, 1.0)
Env 5(Verification)	(1.0, 5.0)	(1.0, −5.0)
Env 6(Verification)	(1.0, 4.0)	(2.0, −5.0)
Env 7(Verification)	(3.0, 2.0)	(−1.0, −4.0)
Env 8(Verification)	(−3.0, 1.0)	(4.0, −1.0)
Env 9(Verification)	(0.0, 7.0)	(1.0, −6.0)
Env 10(Verification)	(9.0, −4.0)	(−9.0, 3.0)

## Data Availability

Not applicable.
